# Dynamic In-Flight Shifts of Working Memory Resources Across Saccades

**DOI:** 10.1037/xhp0000960

**Published:** 2022-01

**Authors:** Rob Udale, Moc Tram Tran, Sanjay Manohar, Masud Husain

**Affiliations:** 1 Department of Psychology, University of Sheffield; 2 Department of Experimental Psychology, University of Oxford; 3 Nuffield Department of Clinical Neurosciences, University of Oxford

**Keywords:** working memory, trans-saccadic memory, encoding, attention, eye-movements

## Abstract

Little is known about how memory resources are allocated in natural vision across sequential eye movements and fixations, as people actively extract information from the visual environment. Here, we used gaze-contingent eye tracking to examine how such resources are dynamically reallocated from old to new information entering working memory. As participants looked sequentially at items, we interrupted the process at different times by extinguishing the display as a saccade was initiated. After a brief interval, participants were probed on one of the items that had been presented. Paradoxically, across all experiments, the final (unfixated) saccade target was recalled more precisely when more items had previously been fixated, that is, with longer rather than shorter saccade sequences. This result is difficult to explain on current models of working memory because recall error, even for the final item, is typically higher as memory load increases. The findings could however be accounted for by a model that describes how resources are dynamically reallocated on a moment-by-moment basis. During each saccade, the target is encoded by consuming a proportion of currently available resources from a limited working memory, as well as by reallocating resources away from previously encoded items. These findings reveal how working memory resources are shifted across memoranda in active vision.

## Public Significance Statement

We continuously make rapid eye movements called saccades to inspect the world around us. A short-term store is needed to hold this information temporarily, to build an internal representation of the visual scene, or guide our actions within the visual environment. It is well established that visual short-term memory is highly limited in capacity, which confronts us with a challenge: When storing information across saccade sequences, how do we reallocate memory resources to new incoming information? Here, we show that resources are dynamically reallocated away from previously fixated items stored in memory to make room for the new saccade target. A large amount of information is stored about this target, even before the saccade is made. Our results suggest a mechanism of how limited memory resources are dynamically allocated in order to support our ability to remember information from the visual scene.

Working memory (WM) is the ability to temporarily hold and manipulate information “in mind.” WM provides the “workbench” for higher cognition, such as thinking, planning, and problem-solving (Hambrick & Engle, 2003). The classic view of WM is that representations are supported by a fixed capacity of slots (Cowan, 2001; Luck & Vogel, 1997), with items that have entered a slot being available for subsequent recall. This view has largely given way to one in which WM representations rely on a limited pool of continuous memory resources (Bays & Husain, 2008; van den Berg et al., 2014). Under the resource view, rather than items being recalled in an all-or-nothing fashion, they can be encoded to varying degrees of precision, with precision depending on the quantity of resource allocated to each item (Bays & Husain, 2008; Wilken & Ma, 2004). It is believed that this mnemonic resource can be flexibly allocated to items. For example, when items are cued as being more relevant to the task, they are recalled much more precisely, indicating that they received a larger share of the resource pool (Allen et al., 2021).

Many aspects of our current understanding of WM have arisen from research using sequences of stimuli. Such serial WM studies, whether in the verbal or visual domain, have led to fundamental findings such as recency and primacy effects, which have been crucial to theories of WM function (Baddeley, 1986). Within the framework of WM resources, it is believed that encoding a sequence relies on reallocation of resources from previously encoded items to items at end of the sequence (Gorgoraptis et al., 2011). There is one key aspect of serial visual WM, however, that has largely been neglected in the memory field. In everyday life, visual information is extracted sequentially as a result of making eye movements across scenes with multiple—simultaneously present—objects (Henderson & Hollingworth, 1998). Most studies of serial visual WM have ignored or tried to control for saccades; for example, by presenting stimuli at fixation (e.g., Gorgoraptis et al., 2011; Matsukura et al., 2007), or by ensuring participants do not move their eyes during simultaneous displays (Bays et al., 2009; Oberauer & Lin, 2017). But such an experimental strategy risks failing to understand fundamental aspects of how WM operates in natural, active vision.

The importance of investigating the impact of saccades for perception has, on the other hand, long been appreciated (Bridgeman, 2010; Deubel et al., 2004; O’Regan, 1992; Rolfs, 2015). For example, it is widely acknowledged that a key challenge facing the visual system is to integrate sequences of limited, temporally discrete inputs into a unified representation of a stable world (Bridgeman, 2011), and to maintain correspondence across the disruption caused by saccades (McConkie & Currie, 1996; Tas et al., 2012). Visual information from the environment has to be extracted with only a limited (foveal) portion of the visual field perceived with great fidelity at any one time. Sampling of the visual scene is interspersed between saccades, during which vision is suppressed, as gaze is directed to inspect new locations. How visual perception is kept stable during such dynamic, intermittent sampling has been the focus of much interest, with many different mechanisms proposed (Bridgeman, 2011; O’Regan, 1992). Additionally, paradigms that have taken into consideration the participant’s natural saccadic behavior have much greater ecological validity than the standard WM paradigms described earlier, because they allow participants to move their eyes in a more natural way akin to how they might in everyday life. These approaches also allow questions to be asked such as how fixation duration and position affects encoding (Irwin & Zelinsky, 2002), how saccades support scene perception (Unema et al., 2005), and how memory and active vision interact (van der Stigchel & Hollingworth, 2018).

Just as utilizing saccadic behavior has been instrumental in understanding visual perception, we believe the same approach will be important for an ecologically valid understanding of visual WM. We argue that, rather than ignoring or inhibiting saccades, we can reach a much more detailed understanding of WM by investigating how it affects, and is affected by our eye movements. There already exist data on this question. For example, saccadic behavior during WM encoding and maintenance can be used to identify the encoding strategy used (Ballard et al., 1995; Meghanathan et al., 2019), or the trade-off between maintenance and further sampling (Draschkow et al., 2021) Additionally, allowing participants to freely refixate the locations of maintained items benefits maintenance, suggesting that saccades are involved in a spatial rehearsal mechanism (Pearson et al., 2014). Finally, Irwin and Zelinsky (2002) presented participants with photographs of scenes containing multiple objects, which they were allowed to view freely. After a brief delay, their memory was probed for one of the items. The authors found performance depended on the number of fixations each item received, and the position in the sequence that each item was fixated, concluding that information about the display accumulates across fixations, but which is limited to approximately five items. One issue with this paradigm is that if encoding relies on a reallocation of resources, the recognition method of probing memory does not provide a continuous measure of the resources allocated to each item within the sequence.

In this paper we attempt to bridge the gap between the literatures that have utilized free-viewing methods and the standard WM paradigms that have traditionally neglected saccades in order to understand the relationship between saccade sequences and WM encoding. The methodology we employ involves gaze-contingent eye tracking so that stimuli can be dynamically extinguished at specific points in a saccade sequence. In addition, we use both direction change identification (Bays & Husain, 2008) as well as analogue, continuous report measures to provide indices of precision of recall (Bays et al., 2009; Wilken & Ma, 2004; Zhang & Luck, 2011). The latter have been particularly instructive in studies of visual WM where stimuli are presented either simultaneously, or sequentially at fixation (Gorgoraptis et al., 2011; Ma et al., 2014).

Because this continuous resource view described previously was largely developed using simultaneously presented stimuli (with saccades being controlled or ignored), it was often assumed that the amount of resource allocated to each item depended on a division of the total available resource (Bays et al., 2009; van den Berg et al., 2012), without accounting for the effect of shifts of attention or saccades. If WM relies on a limited pool of resources, there are a number of ways in which those resources might be allocated across a sequence of saccades. The first, is that resources are withheld until an item is attended. As each item is fixated, a share of the remaining withheld resources are deployed to that item. A drawback of this mechanism is that it relies on knowing how many items are going to be in the sequence, in order to make optimal use of the available resources. It also seems to be an unlikely mechanism in the face of empirical data, as it would not produce observed serial position effects (Gorgoraptis et al., 2011; Kool et al., 2014).

A second mechanism would be to deploy all of the available resources when encoding the first attended item, and when encoding subsequent items, to reallocate a proportion of those resources away from the first item. This makes encoding sequences of any length possible, as a proportion of resources simply needs to be reallocated from already-encoded items to the next one entering WM. Such a mechanism would produce a recency effect. However it would predict that the final item in a sequence would receive the same amount of resource, regardless of the sequence length, because it would receive the same proportion of the total resource capacity.

Empirical studies of serial visual WM using analogue report measures show, however, that recall for the final item appear to depend on the sequence length: the longer the sequence length of items presented consecutively at fixation, the worse the recall precision for the final item (Gorgoraptis et al., 2011). Thus, even the recency effect is modulated by how much previous information has been stored in WM. This raises a third possible explanation: with each new item, a proportion of resources is reallocated from representations already existing in WM, but in addition there is a deployment of currently unused available resources. Our aim was to test this model of dynamic reallocation. By measuring recall error using a continuous scale (magnitude of either localization or orientation error), rather than a binary correct/incorrect outcome, we probed how both visual and spatial WM resources are dynamically redistributed when new information must be encoded on top of items already encoded from previous fixations.

An additional factor that might impact on sequential resource allocation is the effect of attentional shifts before a saccade, as there is known to be a tight coupling between covert attention and eye movements (Casteau & Smith, 2020). Previous research has examined dynamic shifts of covert attention prior to eye movements that results in enhanced perceptual processing of the to-be-fixated item (Hoffman & Subramaniam, 1995; Kowler et al., 1995), finding that resources are obligatorially transferred to the saccade target prior to a saccade (Shao et al., 2010;), conferring a memory benefit for an item at that location (Bays & Husain, 2008; Tas et al., 2016). Those studies demonstrated that presaccadic processing is an important part of encoding information into WM. However, the contents of WM change with every eye movement—as a new item is fixated, resources must be reallocated away from existing representations to the next saccade target. Additionally, individual items within WM be protected from interference if they are placed within the focus of attention (Oberauer, 2002). These previous studies have not yet investigated how presaccadic processing interacts with the changing state of WM with each new saccade. To the best of our knowledge, there is no existing model that accounts for both reallocation of WM resources to the upcoming saccade target as well as how this varies according to saccadic sequence length.

Here, by systematically varying the length of eye movement sequences, we were able to probe changes in memory resources and presaccadic encoding fidelity over time. To anticipate: the results of these six experiments revealed three key findings. First, recall precision for an item in a simultaneously presented array depended upon its position in the fixation sequence, suggesting that WM resources are reallocated from previously fixated items to subsequent ones. Second, recall precision was overall lower for items in longer sequence lengths, regardless of fixation position, further supporting the notion of resource reallocation. Third, by offsetting the display before the final item could be fixated, we found a recency benefit for the final saccade target, despite it having never been fixated before the display was extinguished. Crucially though recall error for this final (unfixated) saccade target was actually worse if participants had previously fixated only one item than if they had fixated a longer sequence of three or four items. In other words, the recency effect for unfixated saccade targets was abolished with short sequences. This finding conflicts with the expectation of greater recall error when more—not fewer—items are stored in WM. All the behavioral findings could, however, be accounted for extremely well by a simple model with a limited WM. Within this model, rather than deploying all resources immediately, a proportion of the available—but currently unused—WM resources are allocated to each fixated item. Before and after each saccade, a process of reallocation also takes place, in which a proportion of resources are transferred from items already encoded in WM to the fixated item. The implication of this model is that, within a sequence of saccades, the total amount of information stored about the environment accumulates over time, and the quantity of information stored about individual objects continuously, dynamically changes with every saccade. This sequential accumulation of information accounted well for serial position effects we observed, as well as the reversal of the recency effect for the unfixated saccade target after shorter sequences. The findings reveal how active working memory mechanisms are across eye movements as we extract information from the visual scene.

## General Method

### Participants

The number of participants tested was chosen using a Bayesian stopping rule: we tested additional participants until the Bayes factor in favor of either the null or alternate hypothesis surpassed 20 for the largest interaction. In total, six experiments were performed. In Studies 1–6 respectively, 21 (ages 18–36, male/female [MF] ratio = .38), 24 (ages 18–35, MF ratio = .33), 23 (ages 18–37, MF ratio = .35), 30 (ages 18–37, MF ratio = .34), 32 (ages 18–35, MF ratio: .6), and 29 participants (ages 20–37, MF ratio = .45:1) took part. All participants reported normal- or corrected-to-normal vision. They were reimbursed £10 (Approx. $12) for taking part. Ethical approval was granted by the University of Oxford’s Medical Sciences research ethics committee.

### Stimuli and Materials

Stimuli presentation were controlled using MATLAB (The MathWorks, Natick, MA) and the Psychophysics Toolbox (Brainard, 1997; Kleiner et al., 2007; Pelli, 1997), using a 21” CRT monitor (resolution: 1,025 × 748), with a refresh rate of 100 Hz. Participant responses were recorded using a standard USB keyboard and mouse. Participants rested their heads in a desk-mounted head rest, 60 cm from the screen. Gaze fixation of the right eye was monitored online at 1,000 Hz using a frame-mounted Eyelink 1000 eye tracker. All stimuli were presented on a uniform gray (RGB: [127, 127, 127]) background. The location memoranda (Studies 1–3) consisted of colored squares (.8° × .8° visual angle), comprising seven colors (white, black, red, green, blue, yellow, cyan), chosen randomly at the start of each trial without replacement. The orientation memoranda (Studies 4–5) were black arrows (RGB: [0, 0, 0]) subtending 1.7° × 3.4° visual angle. Fixation crosses were white (RGB: [255, 255, 255]) subtending 1° × 1° visual angle, appearing 10° left/right from the screen center. The stimuli were presented upon an imaginary circle. The center of the imaginary circle on which stimuli appeared was 3° (3.14 cm, viewed from 60 cm) visual angle from the screen center (randomly left/right), and its radius was 8° (8.39 cm). Stimuli locations were selected by pseudorandomly picking angles between, which were converted to positions on the circle. The position angles were redrawn if any two were within at least 40° of one another. Each experiment began with a nine-point calibration of the Eye-tracker. Participants viewed the stimuli with their heads in a desk-mounted head rest, 60 cm from the display. Stimuli were redrawn if they were less than 45° from one another.

### Detecting Saccades and Fixations

In the following experiments, we employed gaze-contingent eye tracking in order to detect when a memoranda had been fixated, and to turn off the display during the saccade toward the next item after a randomly assigned number of fixations had been made. A fixation was recorded if the participant’s gaze remained continuously within 2° of the target location for at least 150 ms. Study displays remained on until a saccade was detected moving toward one of the items (either the last nonfixated item in Studies 1 and 2, or after having fixated two, three, or four items in Studies 3–5). This saccade was detected if the gaze distance traveled between the current and last sample was greater than 50°/s, and the extended saccade vector was traveling to a point within 1° surrounding the center of any item that had not yet been fixated. In the unlikely event that the saccade vector intersected two unfixated items, the trial was restarted with a new set of randomly drawn stimuli. Throughout data collection, the first author monitored the participant’s gaze position in order to ensure compliance with the task. After the data collection was completed, we assessed the raw gaze data to ensure that the saccades did reliably land on the items detected by the on-the-fly analysis and that the thresholds we used were reliable.

### Analyses

The data from all experiments were analyzed using a Bayesian ANOVA (BANOVA) in JASP (JASP Team, 2021). We report the model-averaged Bayes factors using default priors (fixed effects: .5, random effects: 1, covariates: .354; Rouder et al., 2012).

## Study 1 and 2: Change Identification for Location

First we used a change identification task to examine recall across saccade sequences. Previously, Bays and Husain (2008) conducted an experiment in which participants sequentially fixated items in a simultaneously presented display. As the participants began their saccade toward the final item, the display disappeared, and they were probed on either one of the previously fixated items, or the final saccade target. They found that recall precision for the final saccade was much greater than for any of the previously fixated items, despite not being fixated itself. One likely interpretation of this result is that, prior to each saccade, the next item in the sequences is encoded into WM (or a transsaccadic memory) during a presaccadic covert shift of attention toward the saccade target. Our aim for Study 1 was to replicate this experiment. One potential flaw in its design, however, was that the final item might receive an encoding benefit as a result of the task specifics. For example, the final item was always presented at the center of an imaginary circle, whereas all other items were presented around the perimeter of this circle. Additionally, participants would need to plan their saccades around all other items in the display, but not toward this final item, because the task constrained participants to saccade toward that particular item last. Therefore, in Study 2 the instructions were changed so that participants fixated this central item first. If the concept of presaccadic encoding of an item into memory is correct, we should find a final-saccade recall benefit in both Studies 1 and 2.

### Method

**Figure 1 fig1:**
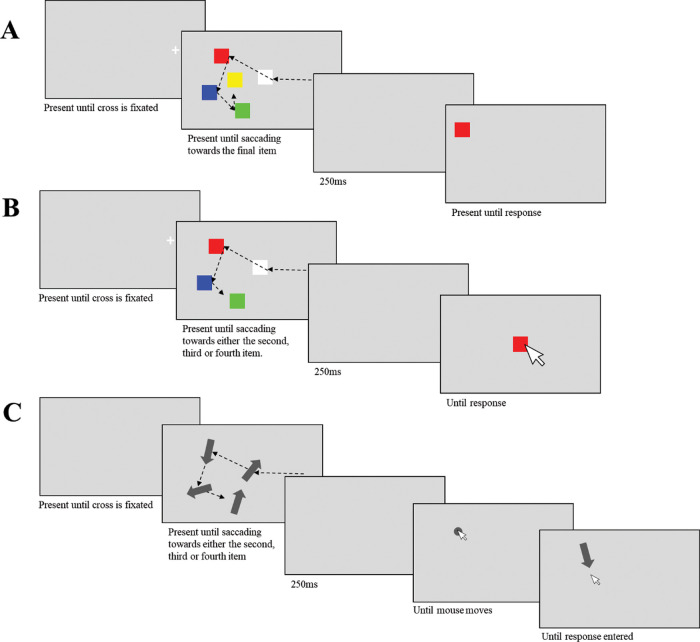
Schematics of Typical Trials Across All Six Studies *Note*. In each task, participants sequentially fixated each item in any order they preferred until the display disappeared, and were then probed on either a fixated item, or the item they were saccading toward. (A) In Study’s 1–2, participants responded by pressing left or right to indicate the direction that the probe moved. We manipulated whether they were instructed to either fixate the central item last (Study 1) or first (Study 2). (B) In Study 3, the participants could fixate the items in any order, and the display disappeared during either the second, third, or fourth saccade. Responses were made by dragging the probe back to its original location. (C) Participants sequentially fixated arrows and reoriented the direction of the probed arrow. We manipulated either the display offset predictability (Study 4), or the total number of items in the display (Study 5). In Study 6, they were able to fixate all items, and the display would disappear after a variable amount of time after fixating the final item in the sequence.

In Study 1 participants were shown four colored squares surrounding one central colored square. They were instructed to sequentially fixate the items, directing their gaze toward the central item last (Figure 1A). This design replicates that of Bays and Husain (2008). As soon as this final saccade was detected, the display was extinguished and replaced with a blank interval for 250 ms. We varied the Probe condition: which item in the saccade sequence was probed, using a change direction identification procedure that parametrically altered the displacement of the probe with respect to the original position of the item. The probed item could appear either .5, 2, or 5° visual angle left or right from its original position (the probe’s location always changed, but varied in degree of change). The maximum possible change magnitude was 5° (5.23 cm). Thus, a study item could appear 11° (11.56 cm) from the screen center, the maximum possible distance for a probed item was 16° (16.86 cm). The screen was 36.86° (40 cm) in width. Thus, it was possible that a probe in the large magnitude change condition, which originally appeared on the outermost edge of the imaginary circle could appear 2.43° (2.55 cm) from the edge. The probe remained on the screen until a response was made. Study 2 was similar, but participants were instructed to fixate the central item first, and to then saccade around the display in any order they preferred. This design allowed us to ensure that any effects observed in Study 1 were not attributable to the fact that the last saccade was, predictably, always to the central item. In all the studies described in this paper, the trial was restarted with new randomly generated stimuli if an item was refixated during a trial. In both Experiments 1 and 2, the trial also restarted with new stimuli if participants fixated the central item out of sequence.[Fig fig1]

### Results

**Figure 2 fig2:**
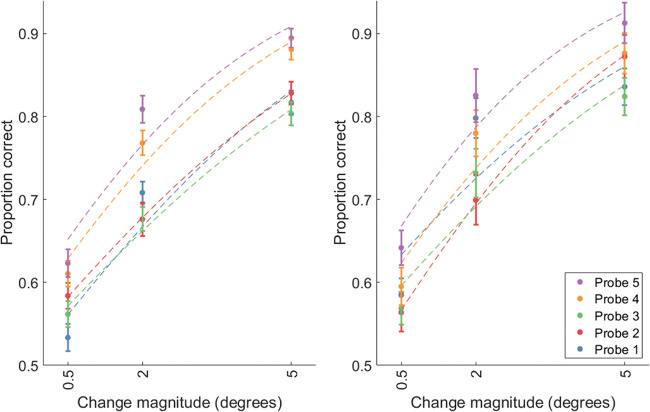
Change Direction Magnitude and Serial Order Memory *Note*. Logistic curves fitted to the mean proportion correct responses as a function of absolute change magnitude and sequence position in Study 1 (left) and Study 2 (right). Dashed curves are logistic functions fitted to the mean of all participants. Error bars depict within subject standard errors calculated using Cousineau’s (2005) normalization method.

Figure 2 shows the proportion of correct responses as a function of sequence position and change magnitude, with a logistic curve fitted to the mean of all participants (Gilchrist et al., 2005; see Figure 2). We conducted a two-way repeated measures Bayesian ANOVA (Rouder et al., 2012) on proportion correct with Sequence position probed (1–5) and Change magnitude (small, medium, large) as independent factors. For this and subsequent analyses, we used the default set of priors in JASP. For ANOVAs the default priors were fixed effects: *r* = .5, random effects: *r* = 1, scale covariates: *r* = .354. For *t*-tests: Cauchy prior with scale = .707. I have reported the model averaged Bayes factors across matched models. The reported Bayes factors are for the model-averaged effects across all models. The analysis was run using JASP (JASP Team, 2021). We found strong support for the main effect of change magnitude (Exp 1: BF_10_ = ∞, Exp 2: BF_10_ = ∞), and sequence position (Exp 1: BF_10_ = 3.29 × 10^8^, Exp 2: BF_10_ = 2.37 × 10^6^). The evidence for the two-way interaction was relatively ambiguous, with the null being somewhat supported in Experiment 1 (BF_01_ = 3.02) and the alternative being somewhat supported in Experiment 2 (BF_10_ = 3.08). Although there was a recency gradient across the sequence of fixations, we wanted to establish if the final item could be recalled much more precisely than the previous items as seen in Bays and Husain (2008). For each magnitude in each experiment, we conducted a paired sample *t* test on accuracy comparing the means of the final item with the penultimate item. Experiment 1 found ambiguous evidence, albeit leaning in favor of the null (small: BF_01_ = 3.98, medium: BF_01_ = 1.66, large: BF_01_ = 3.97). In contrast, posthoc *t*-tests on Experiment 2 found somewhat strong evidence in favor of a difference, particularly for larger magnitude changes (small: BF_10_ = 1.15, medium: BF_10_ = 6, large: BF_10_ = 19.78).[Fig fig2]

**Figure 3 fig3:**
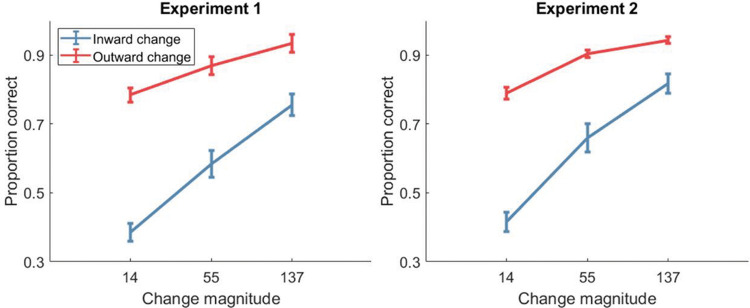
Proportion Correct Change Direction Identification, Split by Whether the Move Was Towards or Away From the Center of Screen

We conducted additional exploratory analysis to identify the effect of the direction of the change relative to the screen (toward vs. away from the center). We found that performance was much worse when the probed item moved toward the center of the screen than when it moved toward the edges, particularly for small magnitude changes (see Figure 3).[Fig fig3]

### Discussion

The results of both experiments show that location recall using a change identification finds a recency effect even when the final item is not directly fixated. Additionally, experiment two showed that, despite the final item not being directly fixated, it can be recalled much more precisely than the item that had just been fixated. However, the magnitude of this effect is much less apparent than shown in Bays and Husain (2008). In Study 1, the final item was always the centrally presented item (replicating Bays & Husain, 2008). However, this benefit was also observed when the final item could be any one of the peripheral items in the display, chosen by the participant (compare the data for Study 1 and 2 in Figure 2). Thus, the final saccade target benefit seems unlikely to be caused by the last item in Study 1 being in a special, predictable location.

Furthermore, we split performance by whether the probe moved outwardly or inwardly (see Figure 3), and found that participants were more sensitive to outward movements than inward ones. Inward movements may be more likely to be confused with other memoranda, because multiple items could potentially appear within the center of the display due to inward movements. In contrast, outward movements are easily identifiable because few items would appear in that region of the display. Some of the increased performance for outward movements may be because participants were peripherally encoding the global spatial layout, and detecting items that fell outside of this. However, because participants were also still able to detect inward movements, suggest that they were encoding specific item-position bindings also.

## Study 3: Variable Sequence Length and Continuous Report for Location

In Study 3, we changed how memory was probed from a change identification to a reproduction task, in which, rather than making a binary decision about the direction of change, participants report the remembered location of the probed item, by dragging the probe with the mouse. This method provides a fine-grained, continuous measure of the quality of the memory representation. The design of Study 3 also aimed to address two further methodological issues associated with Studies 1–2. First, in those experiments the offset of the display occurred during a highly predictable event in the trial: specifically during the saccade toward the final unfixated item. It is possible that, because the participants knew this offset was going to occur, they would encode the final item using covert attention strategically, knowing that they would not be able to fixate it. However, if information about the upcoming saccade target is encoded into WM before every saccade, then we would expect to observe this benefit for every new item to-be-fixated in the saccade sequence. Thus, in Study 3, we varied when the display offset occurred—either during the saccade toward the second, third, or fourth item in the display.

### Method

Participants were shown arrays of four colored squares presented at random locations on a virtual circle. As before, they sequentially fixated the items in any order they preferred. In this study, however, the saccade sequence length varied from trial to trial. On different trials, the display was extinguished (250 ms blank interval) after participants initiated a saccade toward either the second, third, or fourth item (this number was randomly chosen on each trial). After a brief maintenance interval, memory was probed either for one of the fixated items or for the saccade target at offset, which again was never fixated. Note that items in the sequence that were not fixated were not probed, apart from the final saccade target.

The probe display consisted of a colored square reappearing but now always at screen center together with the mouse cursor. Using the mouse, participants moved the probed item back to its remembered location and pressed the space bar to confirm their response (Figure 1B). Thus, we probed by color, the location of one of the memoranda. Each of the nine valid combinations of sequence position offset (where in the sequence the display was extinguished) and sequence position probe (which item was probed) were repeated 40 times, with a total of 360 trials, across 20 blocks.

### Results

**Figure 4 fig4:**
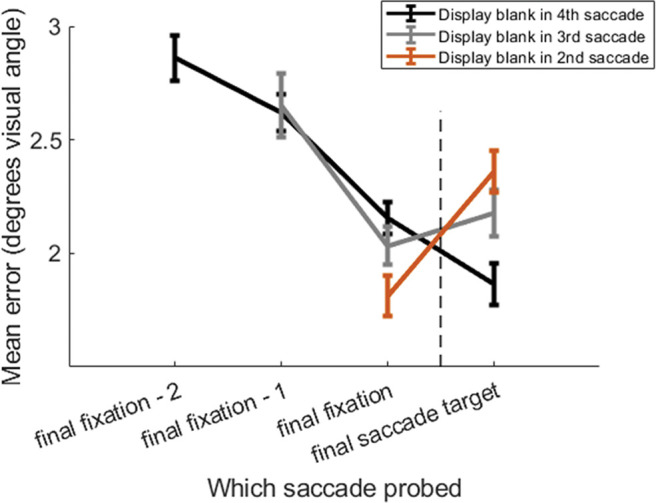
Mean Error as a Function of Saccade Sequence Position and Saccade Sequence Offset *Note*. The vertical dashed line indicates the final saccade when the memory display was extinguished. The probed item could be the target of the final saccade, or the penultimate saccade, or the one previous to that (final saccade—1), or the one before that (final saccade—2). Note how recall error for the final saccade target varies as a function of saccade sequence length, reducing with saccade sequence length.

Mean error (Euclidian distance between presented and reported locations) for each item offset condition was computed (see Figure 4). As in the previous change identification experiments reported in Studies 1 and 2, recall improved for items fixated most recently, but in this experiment there was an important new effect for the (unfixated) final saccade target. When only one item had been fixated and the display was extinguished just as a participant initiated a saccade toward a second item, recall error actually increased for the final saccade target (brown line in Figure 4) compared to the conditions with longer saccade sequences (gray and black lines in Figure 4).[Fig fig4]

We examined how recall error altered between the last item fixated (the target of the penultimate saccade) and the final saccade target (which was never fixated) as a function of the saccade sequence length. A 3 (Offset: Second Saccade vs. Third Saccade vs. Fourth Saccade) × 2 (Probe: Penultimate Saccade Target vs. Final Saccade Target) repeated measures BANOVA was conducted. There was relatively ambiguous-to-weak evidence in support of the main effect of Offset (BF_10_ = 5.01) and Probe (BF_10_ = 7.35). However, there was strong evidence in support for a two-way interaction between Offset and Probe (BF_10_ = 26.81). As the number of previous fixations in the sequence increased, response error for the upcoming, final saccade target reduced. Specifically, performance was worse for the final item (relative to the penultimate item) at set size two, equivalent at set size three, and better at set size four. Posthoc paired sample *t*-tests between the penultimate and final item for each sequence length confirmed this view (Sequence length 2: BF_10_ = 7.06, Sequence length 3: BF_01_ = 2.48, Sequence length 4: BF_10_ = 5.04).

### Discussion

In Study 3, as in Studies 1 and 2, we found that recall error was lower for more recently fixated items. Additionally, recall error was overall higher for items encoded during longer sequences. This is consistent with the notion that some of the resources that are deployed earlier in the sequence are reallocated to encode items presented later in the sequence. However, paradoxically, recall error for the final (unfixated) saccade target in each sequence also decreased as the sequence length increased. This recency effect for the last saccade target was actually abolished and reversed for short saccade sequences. This result contradicts expectations that there would be fewer resources available for the saccade target at the end of a longer sequence compared to a shorter one. It is very different from the findings of a serial WM task that also used continuous, analogue report measures but for stimuli presented consecutively centrally, at fixation (Gorgoraptis et al., 2011). In that study, the last item that had been viewed was always remembered with greatest precision (recency effect) but as sequence length increased, recall precision for the last item decreased. To investigate whether the findings reported here for spatial visual WM (location) also extend to nonspatial visual WM, we next performed a similar study but this time using recall of visual orientation.

## Study 4: Predictable Sequence Length and Continuous Report for Orientation

The primary aim of Study 4 was to see whether it was possible to replicate the effect found in Study 3—that of increasing precision for the final saccade target after increasing sequence lengths in the domain of nonspatial visual WM, rather than spatial visual WM. Therefore, we conducted the same task—sequentially fixating items, turning off the display as a saccade is made toward one of them, and probing memory for one of the items in the sequence. However, participants were now asked to remember and report the orientations of black arrows, rather than the locations of colored squares. We also aimed to address a theoretical issue with this experiment. If the curious effect described in Study 3 arises from reallocation of resources before and after a saccade, is the quantity of resource dynamically reallocated obligatorily fixed, or under some strategic control? To answer this question, we conducted this next study in different blocks. In some blocks, all trials were randomly intermixed, making it impossible to anticipate when the display offset was going to occur. In other blocks, the number of fixations before the display offset was the same for all trials (through which item in the saccade sequence was probed was always randomized). Thus, the offset would be predictable, and if resource allocation is under strategic control, the participants would be able to preallocate resources in anticipation of the memory load at the start of each trial.

### Method

Study 4 aimed to confirm whether the findings of Study 3 were not specific to unpredictable sequence lengths, and location memory. We examined memory for orientation and varied the extent to which participants could predict which saccade initiated the display offset. The method was as in Study 3, except that the memoranda were now dark gray arrows and the probe consisted of a dot appearing in one of the fixated or saccade target arrow locations (Figure 1C). Once participants moved the mouse >.5° on the screen, the dot was replaced by an arrow pointing toward the cursor. They were instructed to reorient the arrow back to its remembered orientation and confirm their response with the space bar.

Furthermore, the task was split into mixed and unmixed blocks. In the mixed blocks, the offset (when in the sequence the display was extinguished) and probe (which item was probed) conditions were randomly intermixed. In the unmixed blocks, the offset condition was kept the same throughout the entire block, but the probe conditions were intermixed. At the start of each block, participants were informed whether the trials would be mixed or unmixed and, if unmixed, how many saccades they would make before the offset. The order of the mixed/unmixed blocks was counterbalanced across participants. Each cell in this design was repeated 20 times, with a total of 360 trials, split into 18 blocks. A schematic of a typical trial in Experiment 4 is shown in Figure 1.

### Results

**Figure 5 fig5:**
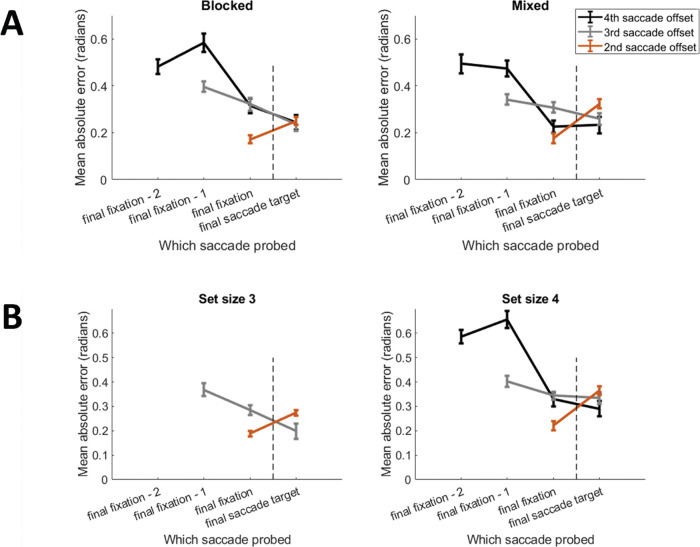
Mean Absolute Error for Orientation in Studies 4 and 5 *Note*. (A) Error as a function of saccade sequence position and sequence offset in Study 4, split by whether the offset was predictable (left) or unpredictable (right). (B) Error across saccade sequences in Study 5, for three (left) or four (right) items in the display.

Figure 5A plots the data from this experiment. A 2 (Predictability: Mixed vs. Unmixed) × 3 (Offset: Second Saccade vs. Third Saccade vs. Fourth Saccade) × 2 (Probe: Penultimate Fixation vs. Final Saccade) repeated measures BANOVA was conducted on the mean absolute error (absolute difference between the presented and reported orientation of the probed arrow). There was strong evidence for a two-way offset and probe interaction (BF_10_ = 40.19), as in Study 3, indicating that the final saccade target was recalled better after longer sequences. However, there was strong evidence against a three-way interaction including predictability (BF_01_ = 19.23), indicating that the relationship was not moderated by predictability. To further explore this, we conducted posthoc paired sample *t*-tests comparing performance for the final fixation and final saccade target at each set size, collapsed across the blocking factor. There was good evidence in support of a difference for the two-item sequence (BF_10_ = 28.8), but not for three- (BF_01_ = 1.2) or four-item (BF_01_ = 3.27) sequences. To summarize, orientation recall for the final saccade target was worse after just a single fixation. However, after subsequent fixations, performance for the final (unfixated) saccade target was at least equivalent, (and potentially better than) the just-fixated item.[Fig fig5]

### Discussion

Study 4 replicated the results of Study 3: recall precision of the final saccade target was as good as the just-fixated item after longer (3+) sequences, and worse after only just a single fixation. This appears to be a change in the representational precision of the saccade target, rather than just a change in the precision of the just-fixated item. Thus, the effect seems to be a general component of visual WM, and not specific to spatial or nonspatial visual WM. Additionally, we blocked the trials so that the number of fixations before the display disappeared was either predictable or unpredictable, manipulating participants’ ability to anticipate and preallocate resources. We found strong support for the null hypothesis regarding the three-way interaction between predictability, offset, and probe. This finding that we observe the same results under both predictable and unpredictable conditions provides two key insights. First, the proportion of resource that is dynamically reallocated with each saccade is obligatorially fixed, and cannot be moderated by strategic control. Second, it rules out the possibility that the participants were using a reallocation rule in which resources are withheld at the start of the trial and the remaining available resources are deployed throughout the sequence without reallocation between items. This is ruled out because, unlike in the unpredictable condition, when the sequence length is predictable, the participants should have been able to optimally reserve resources appropriate for the upcoming sequence length. However, the predictability of the offset did not significantly moderate their performance, suggesting that they did not use this strategy, and therefore must have needed to reallocate resource from previously encoded items during the sequence.

## Study 5: Role of Extrafoveal Encoding in Continuous Report for Orientation

Another potential explanation for the paradoxical effect for the final (unfixated) saccade target is that, as the participant gazes around the display, they build up extrafoveal information about the items to be remembered, without even fixating them. This accumulation of information might lead to better performance for later items without the need for a presaccadic covert shift of attention to the saccade target. To test this idea, we varied the total number of items in the display. Any build-up of information should be limited by the total WM capacity and the memory load. When there are many extrafoveal items, each saccade target should be expected to receive a smaller share of the total resource. Therefore, less extrafoveal information should have accumulated when there are more yet-to-be-fixated items in the display. In Study 5, we used the same design as in Study 4 but removed predictable offset trials. In addition we used a condition in which there were only three items, as opposed to four, present on the screen. Under the extrafoveal buildup hypothesis, the presaccade precision of the third saccade target should be better when there are only three items in the display, as opposed to when there are four.

### Method

As participants move their eyes around a visual scene such as the ones shown in the previous experiments, it is likely that there is a baseline level of encoding of the display through parafoveal vision, in addition to presaccadic parafoveal encoding. To examine the contribution of such “background” parafoveal encoding, we manipulated the total number of items in the display by presenting either three or four items to be remembered. If parafoveal encoding affects saccade target recall, there should be a larger benefit for saccade targets later in the sequence, and items earlier in the sequence may have a benefit when there are fewer items in the display overall. Therefore, in addition to the offset (during which saccade the display blanks) and probe (which item in the saccade sequence was probed) conditions, we varied the set size (three vs. four items). As in other experiments, there were 20 repetitions of each cell in the design.

### Results

Figure 5B plots the data from this experiment. A 2 (Offset: Second Saccade vs. Third Saccade) × 2 (Probe: Penultimate Fixation vs. Final Saccade) × 2 (Set Size: Three vs. Four) repeated measures BANOVA was conducted on the mean absolute error. The set size four, offset four condition was not included in the analysis as there was no comparable condition in the set size three condition. There was strong evidence for the main effects of set size (BF_10_ = 1970.39), offset (BF_10_ = 384.83), and probe (BF_10_ = 434.71). The evidence was ambiguous as to whether there were two-way interactions between set size and offset (BF_01_ = 1.2) or set size and probe (BF_10_ = 2.71). There was very strong evidence for a two-way Offset by Probe interaction (BF_10_ = 1336.57), matching previous experiments. Finally, there was ambiguous evidence for the three-way Set Size × Offset × Probe interaction (BF_01_ = 1.92). Posthoc paired sample *t*-tests showed strong evidence for a difference between the just-fixated item and the saccade target after having only fixated one item (Set size 2: BF_10_ = 49.86, Set size 3: BF_10_ = 84.18). However, after having fixated two items, there was ambiguous-weak evidence in support of the null (Set size 2: BF_01_ = 1.02, Set size 3: BF_01_ = 5.05).

To summarize, there was again greater recall precision for the final (unfixated) target after a longer saccade sequence. However, this was not affected by the presence or absence of a fourth item that would subsequently be saccaded toward. We acknowledge that the ambiguous Bayes factors for the three-way interaction make it difficult to confirm the degree to which parafoveal encoding is playing a role. These results suggest that yet-to-be-saccaded to items consume only a little, if not no, mnemonic resources.

### Discussion

Support for an explanation involving extrafoveal build-up was somewhat inconclusive. Performance for the final saccade target was worse when there were four instead of three items in the display. However, this effect was not supported statistically. The effect of the extra item had little-to-no effect on the direction of the interaction. Therefore, we conclude from this study that, while there may be a degree of extrafoveal build-up, it was insufficient to explain the observed saccade target benefit for longer displays.

## Study 6: Time Course of Memory Resource Allocation

Next we wanted to investigate the time-course of resource reallocation during encoding across saccade sequences. The previous experiments showed, as might be predicted, that the last item that is fixated is remembered best of all the fixated items. If this is due to a reallocation of resources away from items already stored in memory after the final item is fixated, we might expect to observe a decrement in recall for previously fixated items as well as an increase in performance for the final item as a function of the time spent gazing at the final fixated item. Alternatively, if resources are reallocated prior to the saccade, we would expect no difference in recall performance for the final item, regardless of the time that the last item had been fixated. The method of Study 6 was to measure orientation recall error when participants could fixate all items in the display, including the final item. The display disappeared after a variable amount of time (100 ms or 500 ms), or immediately upon fixation of the final item (0 ms offset) —analogous to the condition in our earlier studies in which the final item could not be fixated.

### Method

We used the same design of Study 4 but varied from trial to trial the number of items in the display (one, two, or four) and allowed participants to fixate each item, including the last. The display disappeared once the participants had fixated the final item in the sequence for different intervals (0 ms, 100 ms, or 500 ms). Note that the 0 ms condition is analogous to the condition in our earlier studies in which the final saccade target could not be fixated. Each condition was presented 20 times.

### Results

**Figure 6 fig6:**
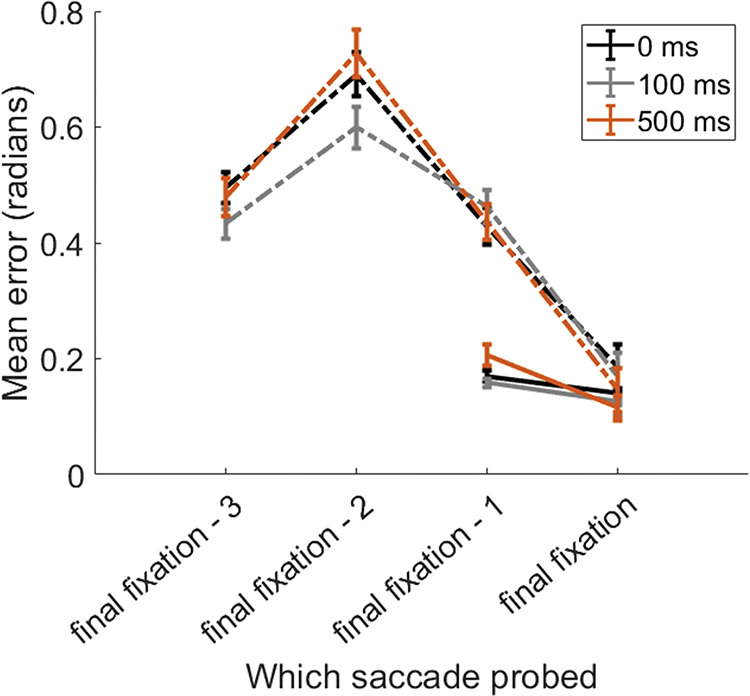
Mean Absolute Error as a Function of Sequence Position, Set Size and Final Fixation Duration *Note*. Error for each sequence position, split by total sequence length (dashed vs solid lines) and presentation time of final item (indicated by color). Note that the 0 ms condition corresponds to the condition where the last item was not fixated because the display was extinguished before the saccade reached this final target.

The results of this experiment are plotted in Figure 6 We conducted a 2 (Set Size: Two, Four) × 2 (Position: Penultimate, Last) × 3 (Duration: 0, 100, 500) Bayesian ANOVA on mean absolute error. There was a strong overall difference between set sizes (BF_10_ = ∞), and position (BF_10_ = ∞) and a two-way interaction between set size and position (BF_10_ = 3.09 × 10^9^). However, there was strong evidence against an effect of duration (BF_01_ = 83.33) or interaction between duration, position, and set size (BF_01_ = 1012.04). These results show that recall precision is best for the final item and therefore would be consistent with memory resources being reallocated across saccades—leading to the effects of sequence position and set size (see Figure 6). However, we found the same recall precision for the final item, regardless of sequence length or fixation time. Additionally, final item presentation time had no effect on precision of older items. Note that this applies also the 0 ms condition in which the final item was never fixated.[Fig fig6]

We have conducted further analyses to understand the correlation between fixation time and memory performance (mean absolute error), split by which sequence position was probed. We found weak negative correlations with ambiguous Bayesian support. However, the correlation was strongly supported for the second item to be fixated (Fixation 1: *r* = −.05, BF10 = 2.7, Fixation 2: *r* = −.08, BF10 = 2,777, Fixation 3: *r* = .01, BF10 = 1, Fixation 4: *r* = −.05, BF10 = .3).

### Discussion

These results are consistent with Studies 1–5, indicating a reallocation of resources from previously fixated items to the currently fixated item. Additionally, the final item was associated with the same error regardless of presentation time (including whether it was even fixated) or serial position. This is in line with the view that it receives a quantum of resource before the saccade is executed, or much less likely, that reallocation occurs instantaneously upon fixation. One potential explanation is that the task demands were sufficiently different from Experiments 1–5 that participants engaged in a different encoding strategy altogether. Previous studies of WM indicate that participants have a large degree of control over their encoding strategies (Donkin et al., 2016). In this case, because they were able to fixate the final item for various lengths of time outside of their control, they may have engaged in a strategy in which all of the encoding was done before the saccade. This of course has implications for models in which presaccadic encoding is an obligatory process (Zhao et al., 2012).

#### A Model of Working Memory Across Saccades

**Figure 7 fig7:**
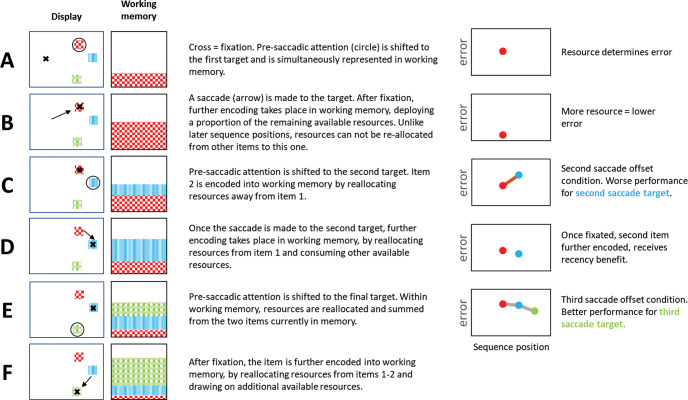
Schematic of a Working Memory Dynamic Resource Allocation Model *Note*. The model simulates the observed data such that memory resource allocation across saccades can lead to more resources being allocated to items later in a saccade sequence. The size of the textured box represents the quantity of resource devoted to each item in WM. A shows the situation when attention shifts from fixation (X) to the target while B shows what happens when the new item is fixated. C and E correspond to the second saccade offset and third saccade offset conditions, respectively. D and F show the second and third saccades. The “error plots” on the right show schematically what the error in recall might be, given the resource devoted to each of the items at different times within the saccade sequence.

We devised an explanatory model to fit data from Study 4 (collapsing across the predictability manipulation) and Study 5, excluding the set size three condition to allow comparison across experiments (see Figure 7). The model assumes that maintaining an item requires a share of mnemonic resource in a limited WM store, with the quantity of resource allocated to an item determining its recall precision (Bays et al., 2009; Wilken & Ma, 2004). At the start of the trial, this store is “empty”. The first attended item is encoded by drawing a proportion of the available resources in this pool. Some resource is allocated prior to the saccade (Figure 7A). Further resource is allocated once the item is fixated (Figure 7B).[Fig fig7]

Encoding subsequent items in the sequence involves two processes. The first is reallocation of resources from existing items in memory to the next target (Figure 7C and E). The second process is deployment of currently unused but available resources—resources that have not yet been allocated to previously encoded items. When each subsequent item is fixated, it receives a proportion of these remaining available resources (Figure 7D and F).

The notion that additional, unused resources can be drawn upon when more items are encoded (in simultaneously encoded “one-shot” displays) is well established empirically (Bays et al., 2009; Oberauer & Lin, 2017; van den Berg et al., 2012). Evidence for this has also been shown in similar sequential fixation tasks (Irwin & Zelinsky, 2002). In the model the total amount of information stored about the external environment sequentially increases. Crucially, as a result, when a proportion of resources are reallocated to the upcoming saccade target, the target receives a large quantity of resource after a longer sequence, than after a shorter one.

The key novel feature captured by this model is that performance for the final saccade target receives a benefit after longer sequences. This is because, after each fixation, a proportion of the currently available (but as yet, unused) resources in WM are allocated to the fixated item. Thus, after each saccade has landed, the total resources that are allocated to any items in memory asymptotically increases toward the capacity limit of the resource pool. Before the next saccade, a proportion of resources are reallocated from items already held in memory to the saccade target. However, because the total quantity of resources that can be reallocated has increased after a longer sequence, the saccade target receives more resources after longer saccade sequences than after shorter ones. This explanation relies on the assumption that there are unused but available resources, which are consumed with each additional item, inspired by experiments that that the total amount of information stored in memory increases with set size (Bays et al., 2009), or with encoding time—up to an asymptote (Bays et al., 2011).

**Figure 8 fig8:**
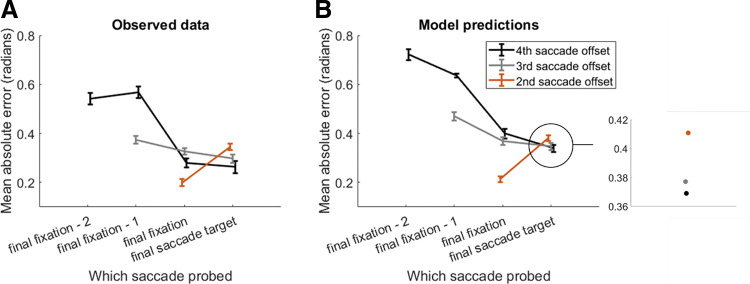
Model Predictions From the Best Fitting (Resource Allocation and Deployment) Model (Right) and Observed Data (Left) *Note*. (A) Data averaged across Studies 4 and 5. (B) Simulated predictions from the resource reallocation with resource deployment model.

**Figure 9 fig9:**
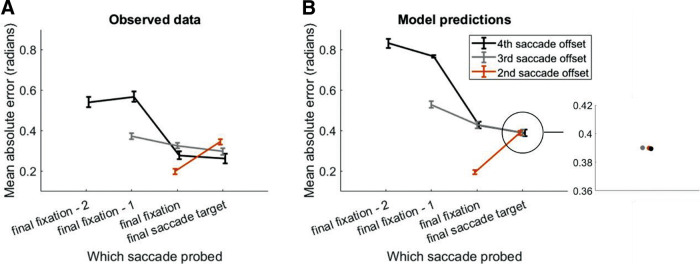
Model Predictions From the Second-Best Fitting (Resource Allocation Without Deployment) Model (Right) and Observed Data (Left) *Note*. (A) Data averaged across Studies 4 and 5. (B) Simulated predictions from the model with resource reallocation and all resources deployed at the start of the sequence.

The model performed better than the same model in which all resources are consumed during the first fixation (ΔAIC = 922.7). Figure 8 shows the close alignment of the predictions from the model in which additional resources are deployed along the sequence with the empirical data collapsed across Studies 4 and 5. Additionally, Figure 9 shows the predictions made from the alternative model in which all resources are deployed at the start of the sequence.[Fig fig8][Fig fig9]

#### Description of Model Fitting

Model parameters were fit to each participant using maximum likelihood estimation using the Nelder-Mead algorithm. Before model fitting, likelihood estimates were calculated for a range of plausible parameter combinations to find suitable initial parameters for the fitting algorithm. We calculated the likelihoods of the response error distribution using the von-Mises distribution, which is the circular analogue of the normal distribution. The von-Mises PDF is given by Equation 1.

$$PDF_{von-Mises}=\;\frac{{\text{e}}^{\kappa \text{ cos}(x)}}{2\pi I_{0}(\kappa )}$$ 1

Where κ is the concentration parameter, x is the error in radians between the presented orientation and the reported orientation, and $I_{0}$ is the modified Bessel function of the first kind of order 0.

Equation 2 shows how the quantity of resource devoted to each item, $r_{i}$, can be calculated for a given κ value. $I_{1}$ is the modified Bessel function of order 1. Values of κ for a given $r_{i}$ were calculated numerically using Equation 2. The value of $\;r_{i}$ indicates the amount of information stored about each item *i* in the sequence, and starts at zero (i.e., no information is known about the stimuli before they appear).

$$r_{i}=\kappa \frac{I_{1}\left(\kappa \right)}{I_{0}\left(\kappa \right)}-\ln\left[I_{0}\left(\kappa \right)\right]$$ 2

To calculate $r_{i}$, we simulated the quantity of information allocated to each item for each time-step (before and after each saccade) according to the rules described below. While in reality, encoding likely takes places in a continuous manner, we assume the simplification of it occurring in discrete steps. This model assumes that representing information in WM relies on two limited pools of mnemonic resources. The quantity of resources devoted to representing an item determines how much information is maintained about it, and subsequently how precisely it is recalled (determined by κ). Each participant has a resource pool, visual working memory with capacity RWM. A value of 0 reflects no information capacity, and larger values reflecting greater informational capacity.

The first item, r1, is encoded and receives a proportion ($R_{wm}\varphi_{init}$) of these resources. Correspondingly, $R_{WM}$ shrinks to $(1-\varphi_{init})R_{WM}$.

Before each subsequent saccade, the saccade target (item i+1) receives a proportion φ of all resources allocated to all j items that have previously been fixated in WM.

$$r_{i+1}=\;\overset{i}{\underset{j=1\;}{\sum }}\varphi \;r_{j}$$ 3

These items lose this same quantity.

$$r_{j}=\;\left(1-\varphi \right)\;r_{j}$$ 4

After the saccade has landed, the fixated item receives additional resources, either directly reallocated from previously fixated items, or through consuming unused available resources

$$r_{i+1}=\;r_{i+1}+\overset{i}{\underset{j=1\;}{\sum }}\varphi \text{′ }r_{j}+\varphi \text{′′}\cdot R_{WM}$$ 5

As before, previously fixated items lose resources

$$r_{j}=\;\left(1-\varphi \text{′}\right)\;r_{j}$$ 6

As does the remaining available WM resource

$$R_{WM}=\;(1-\varphi \text{′′})R_{WM}$$ 7

In summary, the model has five free parameters per participant (360 data points per participant in Experiment 4 and 180 data points per participant in Experiment 5).

## General Discussion

The findings presented here show how serial visual WM operates under more natural conditions than traditionally investigated. Prior investigations have used sequences where each stimulus is presented consecutively, often at fixation, or where saccades were simply ignored. In our studies, the memory set was presented simultaneously, while participants made eye movements from one item to another, analogous to how we extract information about the contents of the visual scene in real life.

Across six experiments, we show that information about the forthcoming saccade target—its location, color, and orientation—is stored prior to fixation. This confirms findings that the saccade target receives a boost in mnemonic resources before saccade onset (Bays & Husain, 2008; Tas et al., 2012). Importantly, and perhaps somewhat paradoxically, recall precision for the (unfixated) final saccade target was significantly better when it was at the end of longer saccade sequences than shorter ones. This benefit was consistent with a model where, rather than all WM resources being initially deployed and subsequently redistributed, additional WM resources are deployed with each fixation, up to an asymptote, along with reallocation from previously encoded items.

This benefit with longer sequence lengths did not depend on strategies that participants may have employed, such as knowledge about when the saccade sequence would be interrupted (Studies 1, 2, and 4). Additionally, it occurred for different feature dimensions being probed (e.g., color in Study 3 and orientation in Studies 4–6), suggesting that it is a general feature of spatial and nonspatial visual WM. The effect is also unlikely to be due to extrafoveal, retinal encoding (Study 5) and is probably better accounted for by the involvement of a presaccadic shift of covert attention (Study 6).

We propose a two-stage model of how resources in WM are handled during sequential encoding across eye movements (see Figure 7). The model assumes that, rather than deploying all resources at the start of the sequence, a proportion of WM capacity is actually left “free” to be allocated later on in the sequence. After encoding the first item in the sequence with a proportion of the available resources, subsequent items are encoded via allocation of resources through two processes. First, before and after each saccade, a proportion of resources are reallocated away from items that are already encoded, and given to the saccade target. Second, once the target is foveated, additional resources—which have thus far been left freely available and not used in representing any of the previously encoded items—are deployed to further encode the target. We believe that both forms of resource allocation are justified. First, resource reallocation from previously encoded items is required to explain the observed serial position and set size effects. Second, we compared our model with one which deployed all available resources at the start of the sequence. This model shows that without resource deployment, the final item in the sequence will always have the same resource quantity, which does not align well with the observed data (see Figure 9).

This deployment of additional resources leads to an accumulation in the total amount of information about the display stored, and in conjunction with the reallocation process, results in the observed recency benefit and set size effects. The apparent paradoxical finding of better performance for the final saccade target after longer sequences can be explained by this two-process model: As the participants initiate a saccade toward the next item, they redirect resources away from previously encoded items toward the target. After longer sequences, there is a greater quantity of resources available that can be reallocated, due to the accumulation of information after each fixation. As a result, the final saccade target receives a boost in performance after longer sequences than shorter ones. An implicit assumption of the model is also that, after each trial, all of the contents of WM are jettisoned, freeing up all of its resources for the next trial.

The broad implication of this model is that, when able to freely view displays such as occurs in real-life settings, the quantity of information stored about each item depends on its position in the saccade sequence, which changes dynamically from moment to moment as the eyes are moved. Thus, models of memory allocation which assume that resources are equally (Bays & Husain, 2008) or stochastically (van den Berg et al., 2012) allocated can be improved by incorporating a sequential reallocation process—even for simultaneously presented information. The effect of fixation sequence may also be a significant contributor to the unequal precision of encoding observed in simultaneously presented displays (van den Berg et al., 2012).

Another implication of our model is that observers do not consume all potentially available resources right from the start of the saccade sequence, so the first item does not receive all of the resources that are available. Rather, each item is allocated only a portion of the total, and other available resources are drawn upon throughout the sequence. There are several possible explanations for this. Study 4 makes it unlikely that resources were strategically withheld for later in the sequence, because the pattern of results were little affected by the predictability of the display offset. It is also unlikely that the memoranda were only partially encoded (e.g., due to short fixation times) because fixation times did not predict recall precision (Study 6).

One possible explanation as to why not all resources are deployed initially might be that there is additional memory load associated with planning a saccade sequence, so as saccades are executed, this memory load naturally decreases, freeing up more resources to encode the targets. However, an alternatively possibility is that deployment of total available resource is a natural consequence of sequential encoding—that additional resources cannot be drawn upon until additional items need to be encoded. Indeed, many established models of serial memory in the context of verbal short-term memory (STM) deploy additional “resources” when new items are encoded due to the way items are represented in these neural network models (e.g., Page & Norris, 1998), and the measured capacity in simultaneously presented “one-shot” visual WM tasks often increases when more item must be encoded (Bays et al., 2009). Perhaps withholding resources for later deployment is a more energy efficient method for allocating resources than initially deploying all resources which must later be reallocated.

Furthermore, Irwin & Zelinsky (2002) also found an apparent accumulation of information over saccade sequences. In their experiment, participants performed a change detection task for items in a simultaneously presented display, which they were able to freely view. They found that recall performance increased with the number of fixations, suggesting a build-up of information over the sequence of fixations, albeit up to a limit of about four–five items. Additionally, consistent with our findings, they found that as the number of prior fixations increased, identification performance increased for unfoveated saccade targets, and decreased for foveated items. One important consideration regarding their method is that participants were able to fixate the memoranda multiple times, and therefore it was not possible to separate encoding due to a presaccadic shift of attention to the saccade target from information about the item encoded during prior fixations. In our study, because the participants saccaded toward each item only once, we could confidently separate the effects of presaccadic encoding from encoding during prior fixations. Furthermore, we also varied set size, which allowed us to measure how much additional information was encoded with each additional fixation.

Our results therefore demonstrate the robustness of these effects in a new paradigm across six new experiments, supporting, and building upon the conclusions of Irwin and Zelinsky (2002). We depart from their conclusions, however, by refining their proposal with a computational model of resource reallocation in which WM is not limited by the number of items that can be encoded, but by the quantity of reallocable resources. A slot-like model as suggested by Irwin and Zelinsky (2002) would not produce the continuous effects observed in our experiments, such as a gradual decline in performance across set sizes. The implication of our model, in contrast to a slot model, is that an arbitrarily long sequence of fixations could hypothetically be encoded, although the memory precision of individual items would be limited by the size of the resource pool, and the dynamics of reallocation across the sequence.

One additional account that might potentially explain these data is the possibility of a separate pool of resources distinct from visual WM—namely, transsaccadic memory (TSM)—that is capable of storing only the currently fixated item and saccade target. If TSM stores only information about two items from before and after a saccade, the first two items in the sequence might be stored there, and encoded with a low degree of precision—explaining why recall for the first saccade target alone is relatively poor. Upon needing to encode longer sequences, WM may need to be engaged, as it is capable of storing multiple items beyond just the first two. As a result, performance for subsequence saccade targets will be better, because they will receive representation in the larger resource pool of WM. This alternative account seems unlikely considering the additional complexity of a model that needs to invoke a two-item limited TSM, and the bulk of evidence suggesting that TSM shares a lot of features with VWM, and that TSM and WM may in fact represent the same construct (Aagten-Murphy & Bays, 2019; Cronin & Irwin, 2018; Irwin, 1991; Hollingworth et al., 2008; Shao et al., 2010;Van der Stigchel & Hollingworth, 2018).

The TSM buffer has been considered to provide correspondence across the disruption to perception during a saccade (Aagten-Murphy & Bays, 2019; Irwin, 1991; Van der Stigchel & Hollingworth, 2018). However whether it is a distinct store from WM is debated (Aagten-Murphy & Bays, 2019; Frost, 2019; Irwin, 1991; Van der Stigchel & Hollingworth, 2018). The results of Study 6 might be accounted for by a separate TSM, because performance for the final item was the same, regardless of fixation time (unlike simultaneously encoded displays, Bays et al., 2011), or the number of items previously encoded (unlike sequential tasks which remove saccades, Gorgoraptis et al., 2011). This suggests that memory precision for this final item was fixed and independent of the quantity of resources held in WM. While the results are consistent with a two-store model, they also fit parameterizations of the single-store model we have proposed, which therefore may be a more parsimonious account of the data.

One of the aims of our approach was to allow participants to encode information in a more naturalistic manner. Specifically, the order in which items were encoded was determined by the participants own choice of saccade sequence. This overcomes the issues we have discussed with ignoring or controlling saccades, or artificially presenting items in a prespecified order. However, in order to probe how resources are allocated throughout the sequence, we needed to artificially blank the study display midsequence. In everyday life we do not usually expect for the visual scene to instantaneously disappear amid visual exploration. Thus our approach deviates from more naturalistic approaches (e.g., Ballard et al., 1995; Draschkow et al., 2021). Future research may need to further explore how findings built upon highly constrained lab-based tasks also apply to more naturalistic gaze behavior.

One potential weakness that may warrant future research is the role of parafoveal build-up across the saccade sequence into WM. Parafoveal information is likely to be encoded along with each fixation—if not for the feature values of parafoveal items, but at least for their spatial configuration. Indeed, analysis of Study 2 suggests that edges of the monitor, which could only have been parafoveally encoded, may have provided some spatial context cues. We attempted to address this question in Study 5, but found insufficient support in either direction for the role (or lack of) parafoveal encoding. The degree to which foveated and parafoveal information contributed to WM representations remains an open question .

To conclude, in everyday life we often need to maintain information across saccades. Here we suggest that this ability relies on a mechanisms of memory allocation in WM with two components: consumption of freely available resources, and reallocation of resources allocated to representations of previously encoded items. Task performance depends on the ability to reallocate resources from stored representations to study targets, as well as to efficiently consume available resources. One aspect we have not yet assessed is whether the rate at which resources are reallocated or deployed reflects optimal tuning of these parameters to minimize energy consumption and maximize performance. We propose that the allocation of memory resources in WM across saccade sequences has been neglected in past research on WM. The results presented here highlight the need to account for covert and overt shifts of attention in encoding memoranda into WM and to account for shifts of resources across saccade sequences. Rather than all items in a simultaneously presented display receiving an equal share of resource (e.g., Bays et al., 2009), recall precision depends on the order in which the probed item was attended in everyday vision. Our model also highlights one important “working” aspect of WM. Rather than passive storage, resources are continuously reallocated on the fly as we engage our eyes with the environment, from moment to moment. Just as our understanding of visual perception has benefited from an appreciation of the role of “active vision” (Findlay & Gilchrist, 2003; Rolfs, 2015), our understanding of visual WM might be improved by understanding its relationship with these ubiquitous eye movements.
